# Researches in Physiology and Pathology

**Published:** 1858-03

**Authors:** 


ART. II.—*Experimental Researches Applied to Physiology and Pathology.* By E. BROWN-SÉQUARD, M.D., etc. etc. New York: H. Bailliere, 1853.

*Researches on Epilepsy; Its Artificial Production in Animals, and its Etiology, Nature, and Treatment in Man.* By E. BROWN-SÉQUARD, M.D., etc. etc. Boston, 1857.

*Essays on the Secretory and the Excito-Secretory System of Nerves, in their Relations to Physiology and Pathology.* By HENRY FRASER CAMPBELL, M.D., etc. etc. Philadelphia: J. B. Lippincott & Co., 1857.

WERE we asked to designate the man who, within the last ten years, has contributed most to our knowledge of the physiology and pathology of the nervous system, we should point unhesitatingly to Brown-Séquard. For, with a constancy worthy of the great subject to which he has devoted himself, he has persevered in his investigations, till he has succeeded in elucidating some of the most abstruse points connected with nervous action, and has thus not only corrected old errors, but has thrown a flood of light upon subjects which, previous to his experiments, were veiled in obscurity. We thus confess at the outset a partiality for our author,—a partiality, however, which, unlike many instances of the feeling, has something good and true to rest upon. We admire his keen sense of the method of conducting experimental investigations, his nice appreciation of the value of his results, and more than these, the genius which directs his observations aright and conquers success. We enter, therefore, upon the duty of noticing his labors with pleasure, and with the satisfaction which all reviewers must experience when they have to deal with that which is valuable and interesting.

The first-named of the volumes before us is a reprint of a series of articles, some of which were published originally in foreign journals, and subsequently contributed in a condensed form to the *Medical Examiner*, and of others, which appeared for the first time in that periodical. As the title indicates, they have reference to the physiology and pathology of the nervous system. They are based mainly upon experimental investigations, or original observations.

The first paper has reference to the "Source of the Vital Properties." After expressing the opinion that every tissue possesses its vital properties in consequence of its peculiar organization, and that in an animal of full development nutrition is the source of its vital properties, he adduces the following experiments in support of this view.

The first series was directed to the investigation of the source of the reflex action of the spinal cord. From these experiments he found that after restoring the circulation to the spinal cord, after its separation from the encephalon, the reflex faculty was also restored, even though this had been exhausted by putting it in frequent and energetic action; and that after dividing the spinal cord in the dorsal region of a mammal, and then killing it by dividing the carotid artery, reflex action was made to reappear, after it had entirely ceased, by injecting blood into the divided vessel. Hence, he very naturally concludes, "that the reflex faculty is a vital property belonging to the spinal cord, and that its source is in the nutrition which maintains the organization of this nervous centre." The tendency of these experiments is, therefore, to confirm the views of Marshall Hall, and others, in reference to the function of reflex action possessed by the spinal cord,—views which, at the present day, are of almost universal acceptance.

The source of the vital property of the motor nerves is next considered. Here it is shown, that although these nerves lose their vital power after separation from the nervous centres, they do so solely because nutrition is then badly performed. In proof of this view the fact is stated, in addition to others, that if, after the motor nerves of a limb have been divided and left dead for half an hour, or even more, the blood, having been stopped, is again allowed to circulate, the vital property of the cut nerves returns; and now in order to produce muscular contraction only a slight compression is necessary.

In the third place, the source of muscular contractility is considered. An experiment of a striking nature is related, which is strongly confirmatory of the opinion advanced, that muscular contractility is not due to nervous power, but to the action of the blood. The sciatic and crural nerves of a Guinea-pig were resected. After ten or twelve days it was ascertained whether they had lost their vitality, and whether the muscles still possessed contractility. These points determined affirmatively, a ligature was put around the aorta, and it was found that after a short time muscular contractility had disappeared and cadaveric rigidity had ensued. An hour afterward the ligature was removed from the aorta, and in ten or fifteen minutes the rigidity had disappeared and the muscular irritability was restored.

Some further experiments relative to the influence of the blood in restoring muscular contractility after it had entirely ceased, are given in Article XXVIII. of the series.

These investigations were instituted upon rabbits and Guinea-pigs, and in two instances on criminals who had been recently guillotined. From them it was ascertained that arterial blood, when injected into the blood-vessels, revived muscular irritability after it had entirely ceased for some hours. In the case of one of the criminals it was re-established seventeen hours and a half after decapitation, and in the other, twenty hours after death, contractility was present in the muscles of the arm.

Other experiments are stated, which are to the same effect. The whole series confirms the result arrived at by Kay, in a similar manner, and, we think, places beyond a reasonable doubt the truth of the view held by Haller, that muscular irritability is, to a great extent, independent of nervous action.

The experiments of Dr. Bennet Dowler, of New Orleans, upon this point, are also exceedingly satisfactory.

The experiments detailed in the third paper, relative to the influence of the nervous system over the functions of organic life, show conclusively that they are performed independently of nervous action. We regard the article relating to this subject as among the most important and interesting in the volume under consideration. Many experiments were made, the results of which are strongly confirmatory of the opinion advanced by the author. For instance, it was found that frogs lived perfectly well, and that digestion continued to be performed with regularity, after extirpation of the medulla oblongata, or of the ganglia of the par vagum. A young cat continued to live for three months after the spinal cord, from the eleventh or twelfth dorsal vertebra, to its termination, had been entirely destroyed. In this animal, during the whole of the above period, the urine continued to be regularly secreted, and was of normal character. This, of course, proves that the separation of the urine from the blood is not due to the action of the spinal marrow; and the results of other experiments are stated, which show that this function is not kept in operation through the agency of the medulla oblongata. Some further researches relative to the continuance of life in animals after the removal of the medulla oblongata, are given in the sixteenth article.

In relation to the mode of action of certain poisons, Brown-Séquard, from numerous carefully couducted investigations, draws the conclusion that the convulsive poisons appear to excite convulsions not by acting directly on the muscles, or on the motor or sensitive nerves, but only on that part of the nervous system endowed with the reflex faculty. Their action, therefore, is indirectly exerted. Brown-Séquard makes the very interesting remark, that he has seen animals die from the effects of strychnine without being at all convulsed. We are not aware that this has been observed by others. The fact is exceedingly important, especially as regards its legal relations, and deserves further investigation. We have killed different animals—dogs, rabbits, birds, frogs, etc.—with strychine, but have always found strong convulsive action produced.

We might refer to other papers in this volume of fully as great interest as those to which we have alluded, but want of space prevents our doing more at present than noticing the concluding article, "On the Cause of the Beatings of the Heart."

In this paper Brown-Séquard advances the opinion that the heart is put in action by an excitant existing in the blood, and that this excitant is carbonic acid.

There are several facts, noticed by observers in this country some years since, which have a bearing upon the cause of the heart's action, and to which it may not be out of place to refer. In 1823, Dr. J. K. Mitchell found that the heart of the sturgeon, after it had ceased to beat, could again be made to pulsate by inflating it with air, and that the heart of the snapping-turtle (*testudo serpentaria*) pulsated strongly when filled in succession with oxygen, hydrogen, carbonic acid, and nitrogen. Professor F. Gurney Smith has also experimented in regard to this subject, and has ascertained that the heart of the sturgeon will continue to pulsate for twenty-two hours after its removal from the body, and that of the snapping-turtle for twenty-six hours. The experiments of Dr. S. Weir Mitchell and Dr. T. H. Bache are also of great interest, as confirming those of Tiedemann, relative to the influence of the atmosphere in exciting contractility in the heart after it had been made to cease by putting it under the receiver of an air-pump and exhausting the air. All these investigations, as well as many others which have been performed in reference to this point, show conclusively that Haller's doctrine, which attributed the exciting cause of the heart's action to the direct agency of the blood acting upon its cavities, is not to be regarded as correct, and gives a good deal of probability to the view advanced in the paper before us.

Brown-Séquard brings forward several facts, which he considers support his hypothesis. They may all be regarded as tending to show that during asphyxia, when, as is well known, there is an accumulation of carbonic acid in the blood, there is an accelerated rate of action of the heart. Any one may convince himself of the truth of this statement by holding his breath for a few seconds. It will be found, that during the suspension of respiration there is a very perceptible increase in the number of pulsations in a given time, and that on readmitting the air to the chest a striking diminution in the number of beats takes place. In fact, (as we have noticed for some years past, and as the investigations of Reid and Mitchell also show,) the action of the heart is invariably slower during inspiration than expiration. This, however, may be due to obvious mechanical causes.

Although we cannot regard the evidence at present before us as definitely establishing the truth of Brown-Séquard's theory, we must admit that it gives it a strong appearance of probability. The function of the heart is so readily affected by nervous agency that it is extremely difficult to investigate it in the living animal with that extreme precision which the subject requires. The least emotion, the slightest attention even to its throbbings, disturbs its regular action, and may, consequently, lead the observer into error. No one, however, has investigated the problem with greater success than Brown-Séquard, and we confidently expect from him more perfect and convincing experiments upon the subject.

The second of the volumes before us contains the results of a series of investigations relative to the artificial production and treatment of epilepsy. The papers composing it were published during the past year in the *Boston Medical and Surgical Journal*, and are now first presented to the profession in a collected form. With three papers contained in the volume we have just noticed, (and which we omitted to consider with the other articles of the series in order to refer to them more appropriately here,) these memoirs exhibit a complete view of the author's researches relative to epileptiform affections.

It is over six years since Brown-Séquard ascertained (*Experimental Researches Applied to Physiology and Pathology*, p. 36) that by making a transverse section of a lateral half of the spinal cord of a Guinea-pig, or a complete section of this organ, an epileptiform affection of an exceedingly violent character was produced after a few weeks. He has since ascertained that the following named injuries of the spinal cord are capable of originating this disease :—

"1st. A complete transversal section of a lateral half of this organ.

"2d. A transversal section of its two posterior columns, of its posterior cornua of gray matter, and of a part of the lateral columns.

"3d. A transversal section of either the posterior columns, or the lateral, or the anterior alone.

"4th. A complete transversal section of the whole organ.

"5th. A simple puncture.

"Of all these injuries, the first, the second, and the fourth seem to have more power to produce epilepsy than the others. The first, particularly, *i.e.* the section of a lateral half of the spinal cord, seems to produce constantly this disease in animals that live longer than three or four weeks after the operation. After a section of either the lateral, the interior, or the posterior columns alone, epilepsy rarely appears, and it seems that in the cases where it has been produced there has been a deeper incision than usual, and that part of the gray matter has been attained. In other experiments (few in number) the section of the central gray matter (the white being hardly injured) has been followed by this convulsive disease. I have seen it but very rarely after a simple puncture of the cord."—P. 4.

The affection generally begins in the course of the third or fourth week, occasionally a few days sooner. At first only the muscles of the face and neck are involved, but the spasms continue to increase in violence and extent, and finally all parts of the body not paralyzed are convulsed.

"Convulsions may come on either spontaneously or after certain excitations. The most interesting fact concerning these fits is that it is possible and even very easy to produce them by two modes of irritation. If we take two Guinea-pigs, one not having been submitted to any injury of the spinal cord, and the other having had this organ injured, we find, in preventing them from breathing for two minutes, that convulsions come on in both; but if we allow them to breathe again, the first one recovers almost at once, while the second continues to have violent convulsions for two or three minutes, and sometimes more. There is another mode of giving fits to the animals which have had an injury to the spinal cord. Pinching the skin in certain parts of the face and neck is always followed by a fit. If the injury to the spinal cord consists only in a transversal section of a lateral half, the side of the face and neck which, when irritated, may produce the fit, is on the side of the injury, *i.e.* if the lesion is on the right side of the cord, it is the right side of the face and neck which are able to cause convulsions, and *vice versa.* If the two sides of the cord have been injured, the two sides of the face and neck have the faculty of producing fits when they are irritated. No other part of the body but a portion of the face and neck has this faculty. In the face the parts of the skin animated by the ophthalmic nerve cannot cause the fits; and of the two other branches of the trigeminal nerve only a few filaments have the property of producing convulsions. Among these filaments the most powerful in this respect seem to be some of those of the suborbitary and of the auriculo-temporalis. A few filaments of the second, and, perhaps, of the third cervical nerves, have also this property of producing fits. In the face the following parts may be irritated without inducing a fit: the nostrils, the lips, the ears, and the skin of the forehead and that of the head. In the neck there is the same negative result when the irritation is brought upon these parts, in the neighborhood of the median line, either in front or behind. On the contrary, a fit always follows an irritation of some violence, when it is made in any part of a zone limited by the four following lines: One uniting the ear to the eye; a second from the eye to the middle of the length of the inferior maxillary bone; a third, which unites the inferior extremity of the second line to the angle of the inferior jaw; and a fourth, which forms half a circle, and goes from this angle to the ear, and the convexity of which approaches the shoulder."

We have indulged thus largely in quotation because we thought it desirable to lay the main points of the author's researches before our readers in his own words. It will be seen how very important the views are which he enunciates, and how surely they rest upon facts. The connection of epilepsy with disease or injury of the spinal cord is distinctly pointed out, and is deserving of attentive consideration, both as regards the pathology and treatment of the disease.

The fact that this connection has not been more frequently remarked is probably due to the circumstance that the spinal cord is so infrequently examined after death, and to the difficulty which has hitherto existed of detecting certain radical alterations of the substance of this organ. Several very striking instances are however adduced, to show that a relation between disease or injury of the spinal cord and epilepsy does exist, and from these cases, and his own investigations, Brown-Séquard draws the following conclusions:—

"1st. There cannot be any doubt that in animals certain injuries to the spinal cord frequently produce an epileptiform affection, if not true epilepsy.

"2d. That in man there are a great many cases which seem to prove that alterations of the spinal cord may cause epilepsy."

Attention is called to the analogy existing between the aura epileptica in man, and the peculiar excitation produced by pinching the skin of the face of animals whose spinal cords have been injured. It would appear that in these animals the convulsions were the result of reflex action, and from a number of cases subsequently quoted it seems that the same explanation of their cause is applicable to man.

While insisting upon the probability that in man epilepsy may be dependent upon alterations of the spinal cord, Brown-Séquard denies the existence of any distinct species of this disease as arising from such affections, and after stating the main points of several of the more prominent theories of epilepsy, especially those of Todd and Marshall Hall, and enlarging somewhat upon their deficiencies, proceeds to give his own hypothesis. This theory is not proposed as complete, but simply as aiding in the elucidation of some of the principal questions connected with the disease under consideration. We will endeavor in few words to lay a summary of his views before our readers.

The brain is not essential to the production of epileptiform convulsions, for after its removal fits may be caused in animals whose spinal cords have been injured, with as much ease as before, by pinching the skin of the face or neck. Slight convulsions may also be induced by the same means, if, in addition, the cerebellum, and even the whole basis of the encephalon, except the medulla oblongata and pons varolii, be taken away. It results, therefore, that the epilepsy of the animals submitted to experiment had its seat either in the pons varolii, the medulla oblongata, the spinal cord, or in all three of these organs together. Facts, however, are not wanting to prove that epilepsy does not always reside in these parts collectively, or in any one of them, and, consequently, that irritation or injury of these organs may exist without epilepsy necessarily ensuing.

"If we neglect the nature of the convulsions, and take notice only of the parts of the body where they first occur, we arrive at the conclusion that the seat of epilepsy is very variable. Usually, however, the first spasmodic contractions occur in the muscles of the larynx, of the neck, of the eyes, of the chest, of the face, and in the blood-vessels of the brain proper, as we will show hereafter; and as these parts are animated by nerves coming from the encephalon, and from the upper parts of the spinal cord, it seems that the seat of epilepsy is in some of these parts, if not in all. But the seat of this disease may be in other parts of the spinal cord, as seems to be proved by the production of the first spasmodic contractions in one of the limbs, either the inferior or superior. After the first spasms, all the muscles of the body may be attacked with convulsions; so that if we take notice of the loss of the actions of the brain proper, there is ground for thinking that the seat of the disease is both in those parts of the cerebro-spinal axis, where reside the faculties of perception and volition, and in those endowed with the reflex faculty; but this view is right only in appearance. We have shown already that the loss of perception and volition does not prove that epilepsy has its seat in the brain proper; we will try in a moment to show the great probability that a contraction of the blood-vessels of the brain proper, due to an irritation of their nerves in the spinal cord and medulla oblongata, causes the loss of the cerebral faculties, and, as regards the increase of the reflex faculty, we will show that a partial and a local increase is sufficient for the production of fits."

In the opinion of Brown-Séquard, epileptic fits always result from an excitation of the cerebro-spinal axis, though this excitation may arise from chemical and physiological changes occurring in the elements of the nervous centres, in consequence of bad nutrition and other causes.

As an irritation of the simple direct motor side of the cerebro-spinal axis cannot give rise to general convulsions, the convulsive movements which result from direct excitations of the nervous centres, as well as those which ensue upon irritations coming from peripheric nerve-fibres, should be regarded as reflex. "The so-called centric and eccentric causes of excitation of epileptic fits both act on or through the sensitive or excito-motory side of the cerebro-spinal centres, and consequently both act on the reflex faculty of these centres, so that they both ought to be called reflex excitations."

After some further remarks relative to the distinction between *reflex force* and *reflex excitability*, and in regard to a *special kind of excitation* acting on the nervous centres, the opinion is expressed that there are these three distinct causes for the production of a fit; that the first two are the more frequent, and perhaps the first of these two, viz. *reflex excitability*, is essential.

The application of these views to the explanation of the several phenomena of an epileptic convulsion, is next dwelt upon. The arguments adduced are very much to the point, and deserve attentive consideration.

The following table, in which the principal phenomena of a fit, and the immediate causes giving rise to them, are concisely stated, is given. A glance is sufficient to explain it:—

| CAUSES. | EFFECTS. |
| --- | --- |
| "1. Excitation of certain parts of the excito-motory side of the nervous system. | "1. Contraction of the blood-vessels of the brain proper, and of the face and tonic spasm of some muscles of the eye and face. |
| "2. Contraction of the blood-vessels of the face. | "2. Paleness of the face. |
| "3. Contraction of the blood-vessels of the brain proper. | "3. Loss of consciousness, and accumulation of blood in the base of the encephalon and in the spinal cord. |
| "4. Extension of the excitation of the excito-motory side of the nervous system. | "4. Tonic contraction of the laryngeal, the cervical, and the expiratory muscles, laryngismus and trachelismus. |
| "5. Tonic contraction of the laryngeal and of the expiratory muscles. | "5. Cry. |
| "6. Further extension of the excitation of the excito-motory side of the nervous system. | "6. Tonic contractions extending to most of the muscles of trunk and limbs. |

| | |
|---|---|
| "7. Loss of consciousness and tonic contraction of the trunk and limbs. | "7. Fall. |
| "8. Laryngismus, trachelismus, and the fixed state of expiration of the chest. | "8. Insufficient oxygenation of the blood, and general obstacle to the entrance of venous blood in the chest, and special obstacle to its return from the head and spinal canal. |
| "9. Insufficient oxygenation of the blood, and many causes of rapid consumption of the little oxygen absorbed, and detention of venous blood in the nervous centres. | "9. Asphyxia. |
| "10. Asphyxia, and perhaps a mechanical excitation of the base of the encephalon. | "10. *Clonic convulsions everywhere,* contractions of the bowels, of the bladder, of the uterus, erection, ejaculation, increase of many secretions, efforts at inspiration. |
| "11. Exhaustion of nervous power generally, and of reflex excitability particularly, except for respiration. Return of regular inspirations and expirations." | "11. Cessation of the fit; coma or fatigue; headache; sleep." |

It is well known that after frequent attacks of epileptic convulsions various disorders of the mind and senses are produced. These are due to the diminution in the amount of blood circulating through the brain in the beginning of an attack of epilepsy, the circulation of black blood through the nervous centres, and pressure upon the encephalon and spinal cord by the accumulation of blood in their vessels.

The *treatment* of epilepsy it is proposed to consider more fully at another time. A few propositions are, however, announced, which consist, when the disease is of peripheric origin, in recommendations for the interruption of the venous connection between the part where the aura originates and the nervous centres, and counter-irritation in the neighborhood of this part; application of moxas along the course of the spinal cord; modification of the nutrition of the nervous centres by medicines which act on the blood-vessels, such as strychnia, atropia, ergot, etc.; trepanning in certain cases; cauterization of the mucous membrane of the larynx; hygienic measures, etc., are also advised. The apparent connection existing between epilepsy and intermittent fever is pointed out, and the generation of this latter disease, with a view to eradicate the former, is recommended. During the fit the best means to prevent or diminish asphyxia are the dashing of very cold water in the face, and the inhalation of chloroform.

We have thus endeavored to present an outline of Brown-Séquard's researches into the physiology and pathology of the nervous system,—an outline which any one who refers to the works in which they are contained will discover is very incomplete. Though some of his views may be incorrect, they are founded on actual experiment and observation, and are, therefore, entitled to a degree of consideration which possibly they might not otherwise deserve. Those of our profession who, feeling how much remains to be done in physiological science before medicine can pretend to certainty, will peruse these volumes with interest, and with the conviction that the specialties considered could not have fallen into more able and energetic hands.

The work of Dr. Campbell, which we now propose to consider, consists of three papers contributed by him to the *Transactions of the American Medical Association*, and a letter to Dr. Marshall Hall in relation to Dr. Campbell's claim to priority in the discovery of and naming the excito-secretory system of nerves. They are now, for the first time, published in a collected form.

The first essay is devoted to the consideration of the nervous system in febrile diseases, and is intended as an introduction to the more elaborate paper which follows it, entitled "An Inquiry into the Nature of Typhoidal Fevers." It was submitted to the Association as a "partial report" upon the subject, from the committee of which Dr. Campbell was chairman.

In this paper, all fevers are arranged into two grand divisions, corresponding to the two grand divisions of the nervous system.

1st. The cerebro-spinal fevers: under which head are embraced all paroxysmal fevers.

2d. The ganglionic fevers: comprising all continued fevers.

Dr. Campbell contents himself at present with merely announcing this classification, and offers no evidence in support of his views. He, however, alludes to the blending of the types of fevers as being easily explainable by his theory. For this purpose, he assumes the propagation of the irritation originally located in the spinal cord, and inducing a paroxysmal fever, to the ganglionic system, and thus giving rise to a continued fever, and *vice versâ*.

Although there is a considerable amount of plausibility in this hypothesis, it is at present entirely unsupported by facts. We trust, however, that Dr. Campbell will investigate the subject more thoroughly, and present us a well digested theory based, in part, at least, upon original observations and experiments.

In the second paper, the title of which we have just mentioned, Dr. Campbell enters more fully into the subject, and has really produced an elaborate essay. Beyond the hypothesis advanced as to the pathology of typhoidal fevers, and which is almost entitled to be considered as original, the whole paper is, however, a *résumé* of the observations of others. We must dissent *in toto* from the opinion enunciated by Dr. Campbell relative to original investigations. He appears to think that by studying the recorded observations of others truth is arrived at with greater certainty than by investigating phenomena ourselves. We think this is a great error—one that, if acted upon, would do much to retard the progressive advancement which so especially marks the science of the present era. The discovery of a law probably requires a higher degree of intellectuality than a facility for observing tangible facts or for performing experiments. Yet we find that almost all great laws and sublime truths were established by those who possessed these qualifications in an extreme degree. A Newton, a Kepler, and a Franklin, would probably have remained in their original obscurity if they had studied only the observations of others. We think, too, that error is much more apt to ensue in the deduction of conclusions, if original research be neglected. Dr. Campbell rejects experiment for the assumed reason that we are too apt to distort our facts to suit our theories. This is doubtless true of some; but those who would deal thus with their own facts, would certainly not be less scrupulous with the facts of others. Besides, arguing upon Dr. Campbell's presumption, how do we know that the observer whose investigation we refer to, and upon which we build our hypothesis, may not himself have been writing with a view to the establishment of a favorite doctrine.

We think, therefore, that experiment, and the promulgation of laws and truths deduced therefrom, are best when connected in the same individual; and that, *cæteris paribus*, he is the best law-giver who possesses the greatest amount of self-acquired experience.

The first thirty-four pages of the essay before us are taken up with descriptions of the symptoms and anatomical lesions observed in the typhoidal fevers. These are very carefully expressed, and evince a great deal of research and study of the best authorities on the subject. We do not know where there is contained, in as small a compass, so complete a history of what has been done on the subject, and so good an epitome of the views entertained by those who have given it most attention.

The next eight pages are devoted to the anatomy and physiology of the sympathetic nerve. This, like the preceding part, will be found to contain the substance of what is known upon the subject.

Eleven pages are given to the pathological deductions from the facts previously stated. The most important of these is, "that typhoidal fevers result from alterations in the condition of the ganglionic nervous system." An opinion is also expressed, that typhoid and typhus are merely different types of the same disease located in different parts of the ganglionic system.

As we before remarked, we regret that Dr. Campbell has not presented us with a series of well-conducted experiments, or original observations of post-mortem appearances, in support of his theory. Without these, his views, however reasonable, cannot be accepted as demonstrated truths, but must be placed by the side of the numerous other unsubstantiated hypotheses which cumber the archives of science.

We come next to consider the most important paper in the volume, viz. the essay on the excito-secretory system of nerves. To this memoir one of the prizes of one hundred dollars was awarded by the American Medical Association, at its last meeting.

In the first place, the pre-eminent value of observation over experiment is asserted. We have already noticed Dr. Campbell's views on this point; but, while differing with him in the main, we do not wish to be understood as attaching an undue estimation to experiment. Without a sufficient acquaintance with the subject to be investigated, or care in the process itself, experimental research is worse than useless; for from it erroneous deductions may be drawn which years will not rectify. We do claim, however, that a good experimenter is almost invariably a good observer; and that observation is never more valuable than when going hand in hand with experiment.

Our readers will, we think, be unprepared for the opinion of Dr. Campbell, that there is already a sufficient accumulation of facts in physiological science, and that nothing remains to be done but the arrangement of these facts into one harmonious system. We are sure Dr. Campbell enunciated this view without due reflection, and not from an insufficient acquaintance with the present state of physiology.

We will not dwell upon the numerous facts brought forward to show the intimate connection existing between the ganglionic system of nerves and secretion—a connection which has gained for the sympathetic the very appropriate name of secretory. Dr. Campbell appears to have well considered and studied this portion of his subject, and good use is made of the material at his disposal. The experiments of Magendie and others relative to the nutritive function of the fifth pair of nerves, are reviewed at length, and their application to the views held by the author is very appropriately made.

The excito-secretory function of the sensory system of nerves is next considered. This is Dr. Campbell's great discovery. Although others had previously noticed manifestations of this power, Dr. Campbell has undoubtedly the merit of having first viewed it in its true light. The importance of this discovery can hardly be over-estimated, and it appears strange, as is the case after every valuable revelation, how it could so long have been overlooked. We have inquired most carefully into all the facts and observations relative to it, and we have pleasure in stating that we have nowhere found the excito-secretory system of nerves distinctly pointed out till Dr. Campbell commenced his essays on the subject. We have no sympathy with those who, having years since made use of some indistinct expressions admitting of almost any construction, follow in the wake of him who states his ideas with clearness and precision, and endeavor to deprive him of the credit which is his due.

We quote the following passage from Dr. Campbell's essay, in illustration of his view, and as clearly setting forth his discovery:—

"The cerebro-spinal system has been subdivided into two portions, viz. the nerves of sensation, and those presiding over muscular action. A *relation* has been discovered to subsist between these two portions of the nervous system, by virtue of which the sensory nerves have been found to act as excitors to the motory; and hence this system has been termed by Dr. Marshall Hall the *excito-motory* system of nerves. Now, as we hope to set forth more fully in the present discussion, these same sensory nerves are not only excitors to the *motory* system, but under certain circumstances most of them sustain an analogous relation to the *secretory* nerves, exciting them and modifying their action, diminishing, increasing, and altering the secretions according to the extent and character of the excitation applied. It will be our object, then, to show that the sensory nerves, or at least some of them, sustain to the other two portions of the nervous system a double relation; first, excitors to the motory system, giving rise to the excito-motory system described by Dr. Marshall Hall, in 1837; and secondly, excitors to the *secretory* system, resulting in the excito-secretory system, enunciated first in this country in the year 1850; and which second system Dr. Marshall Hall did not appear anywhere to recognize until the present year 1857."

In the ensuing pages of the essay, Dr. Campbell abundantly establishes the existence of the excito-secretory system of nerves. He seems to be fully aware of the great value of his discovery; and the relations of the system which he has pointed out, to physiology and pathology, are ably and clearly shown. We are sorry we cannot follow Dr. Campbell in his observations. We must, however, content ourselves by commending his essay to our readers, as containing much interesting and valuable matter.

We cannot but regret that Dr. Campbell, by neglecting experiment, lost the brilliant opportunity—which Bernard knew so well how to improve—of demonstrating his discovery to the world. Had he been more active in this respect, no doubt could possibly have arisen as to his claim to priority; and he would not have been obliged to make the admission that he had, in no instance, tested the chemical properties of the urine of teething children. The suggestion, however, which he makes, that in cases of an increase in the quantity of urine from this cause, it will probably be found to contain sugar, we have recently had an opportunity of verifying, in a child now under our notice.

The letter to Dr. Marshall Hall hardly calls for any special notice at our hands, especially as Dr. Campbell's claim to priority in his discovery has been so generally recognized, and by no one more fully than by Dr. Hall himself. We therefore bring our article to a close. In the words of Dr. Hall, "There is in the excito-secretory function, as applied to pathology, an ample field of inquiry for his [Dr. Campbell's] life's career, and it is indisputably his own. He first detected it, gave it its designation, and saw its vast importance." May we not hope that Dr. Campbell, by original observations and experiments, will make this subject so inseparably connected with his name, that none in future will presume to question his claim.

